# Risk of hypovolemia associated with sodium–glucose cotransporter-2 inhibitors treatment: A meta-analysis of randomized controlled trials

**DOI:** 10.3389/fcvm.2022.973129

**Published:** 2022-11-14

**Authors:** Xi Rong, Yawen Zhu, Bo Wen, Kai Liu, Xinran Li, Qiling Gou, Xiaoping Chen

**Affiliations:** ^1^Department of Cardiology, The Affiliated Hospital of Southwest Medical University, Southwest Medical University, Luzhou, China; ^2^Department of General Practice, West China Hospital, Sichuan University, Chengdu, China; ^3^dMed Biopharmaceutical Company Limited, Shanghai, China; ^4^Department of Cardiology, West China Hospital, Sichuan University, Chengdu, China

**Keywords:** sodium-glucose cotransporter-2 inhibitors, adverse event (AE), volume depletion, meta-analysis, RCTs, hypovolemia

## Abstract

**Aim of the review:**

To assess the risk of hypovolemia for sodium–glucose cotransporter-2 (SGLT2) inhibitors treatment.

**Method:**

A systematic literature retrieval was performed in PubMed, Embase, Cochrane Central Register of Controlled Trials (CENTRAL), Web of Science, and Scopus from inception up to 4 October 2022, Data for study characteristics and outcomes of interest were extracted from each eligible study. Risk ratios (RRs) with a 95% confidence interval (CI) for hypovolemia were calculated using a random-effect model.

**Results:**

A total of 57 studies (*n* = 68,622) were included in our meta-analysis, with a result of 1,972 hypovolemia incidents (1,142 in the SGLT2 inhibitors group and 830 in the control group). The pooled RR was 1.12 (95% CI: 1.02–1.22). It is evident that receiving SGLT2 inhibitors increased the risk of hypovolemia. When stratified by category of SGLT2 inhibitors the result was consistent; when the subgroup was analyzed by age, the pooled RR was 1.07 (95% CI: 0.94–1.23) in patients aged ≥65 years and 1.14 (95% CI: 1.02–1.28) in those aged <65 years. When comparing the baseline estimated glomerular filtration rate (eGFR) of less than or equal to 60 mL/min/1.73 m^2^ with a baseline eGFR greater than 60 mL/min/1.73 m^2^, the pooled RR was 1.21, (95% CI: 1.00–1.46) and 1.08, (95%CI: 0.98–1.20), respectively.

**Conclusion:**

Our meta-analysis has demonstrated that SGLT2 inhibitors increased the risk of hypovolemia in patients with Type 2 Diabetes Mellitus (T2DM). It is necessary to pay attention to the risk of hypovolemia associated with SGLT2 inhibitors, especially in older individuals and those with moderate renal impairment.

**Systematic review registration:**

[https://www.crd.york.ac.uk/prospero/], identifier [CRD42020156254].

## Introduction

Type 2 diabetes mellitus (T2DM) is an important health problem worldwide, which is characterized by insulin resistance, β-cell dysfunction, and impaired glucose tolerance (1). The prevalence of diabetes has been increasing dramatically. It is estimated that the overall prevalence of adult diabetes patients was 10.9% in China (2). Diabetes will be the 7th leading cause of death in 2030 (3). Optimal control of plasma glucose is the crucial treatment for T2DM (4).

Sodium–glucose cotransporter-2 (SGLT2) inhibitors are a new class of drugs, which improves glycemia by enhancing glycosuria, subsequently reducing blood pressure by osmotic diuresis and natriuresis (5). There are multiple large-scale randomized control trials demonstrating that SGLT2 inhibitors had a salutary effect on the cardiovascular-renal outcomes, especially on heart failure (6). Although SGLT2 inhibitors exhibited promising potential value in treatment for type 2 diabetes mellitus (T2DM) with cardiovascular-renal comorbidities, the potential adverse events (AEs) related to osmotic diuresis such as hypovolemia should not be neglected. Moreover, older individuals, those with moderate renal impairment, and those aged ≥65 years are susceptible to adverse events related to hypovolemia.

As for the above reasons, our systematic review targeted to investigate the hypovolemia incidents related to SGLT2 inhibitors by meta-analyzing, and we also performed subgroup analysis depended on the category of SGLT2 inhibitors, patients’ age, and baseline estimated glomerular filtration rate (eGFR) to evaluate whether the risk of hypovolemia could be affected by clinical variables.

## Materials and methods

### Literature search

We performed a systematic and comprehensive literature search in PubMed, Embase, Cochrane Central Register of Controlled Trials (CENTRAL), Web of Science, and Scopus from inception up to 4 October 2022. And we adhered to the 2020 PRISMA (Preferred Reporting Items for Systematic reviews and Meta-Analyses) statement in conducting this study and reporting the results (Supplementary material 1). The search strategy combined the Medical Subject Heading and the text words canagliflozin, dapagliflozin, empagliflozin, ipragliflozin, remogliflozin, ertugliflozin, sergliflozin, luseogliflozin, Sotagliflozin, Tofogliflozin, Sodium glucose co-transporter, SGLT2, SGLT-2, and SGLT 2 (Supplementary material 2). These terms were adjusted to conform with the searching principle of each database; citations without any limits were searched. This systematic review was registered in PROSPERO (CRD42020156254).

### Study selection

Two authors (Xinran Li and Qiling Gou) independently reviewed all relevant studies according to prespecified criteria. Inclusion criteria were: (a) RCTs reported in the English language and included adult patients with T2DM; (b) SGLT2 inhibitors compared with placebo or active comparator; (c) duration of follow-up of at least 12 weeks; and (d) the hypovolemia adverse events, which were investigated using a pre-specified list of Medical Dictionary for Regulatory Activities (MedDRA) preferred terms to identify events of hypotension, dehydration, or hypovolemia in the database (Supplementary material 3). Data from completed published manuscripts were considered for inclusion in this analysis.

### Data extraction and validity assessment

Two researchers (Xinran Li and Qiling Gou) independently screened and extracted the data using a previously defined standardized Microsoft excel sheet; the following information was extracted from each eligible trial: first author, year of publication, trial identifier, study duration, intervention drug, control drug, sample size, patients characteristics, duration of T2DM, and incident of hypovolemia events. These data were further examined by another investigator (Xi Rong), and any discrepancies were resolved by discussion. If hypovolemia events were not reported in the published paper, then these data were instead extracted from the trial register website. If the trial register website also did not provide the data on hypovolemia events, we attempted to contact the author to get the data. Two reviewers independently applied Cochrane risk-of-bias tool (7) to assess the quality of included RCTs based on the following domains: random sequence generation, allocation concealment, blinding of study participants and personnel, incomplete outcome data, selective reporting, and other biases.

### Data synthesis and statistical analysis

Most of the analyses were performed by using RevMan (version 5.3.5; Cochrane Collaboration). For dichotomous data, risk ratios (RRs) and 95% confidence interval (CI) were calculated to appraise the risk of hypovolemia with SGLT2 inhibitors treatment. Furthermore, subgroup analyses were conducted on the category of SGLT2 inhibitors, patients’ age, and baseline estimated glomerular filtration rate (eGFR) to evaluate whether the risk of hypovolemia could be modified by clinical variables. Sensitivity analysis was assessed by omitting one study at a time and re-estimated the combined RR for the remaining studies yielding consistent results to determine whether the result of the original analysis was robust. The Chi-square test (χ^2^) and *I*^2^ statistics were used to assess heterogeneity, Heterogeneity was assessed as low, moderate, and high with *I*^2^ values of 25, 50, and 75%, respectively. A random-effects model was adopted if there was evidence of statistical heterogeneity or clinical diversity (*P*<0.01, *I*^2^>50%); otherwise, a fixed-effects model was used if there was no statistical significance of heterogeneity (*P*>0.01, *I*^2^<50%). The presence of publication bias was evaluated by visual inception for funnel plot asymmetry; Begg’s test and trim-and-fill method were also performed using STATA (version15.0; STATA software) to assess publication bias.

## Results

### Eligible studies and characteristics

**FIGURE 1:**
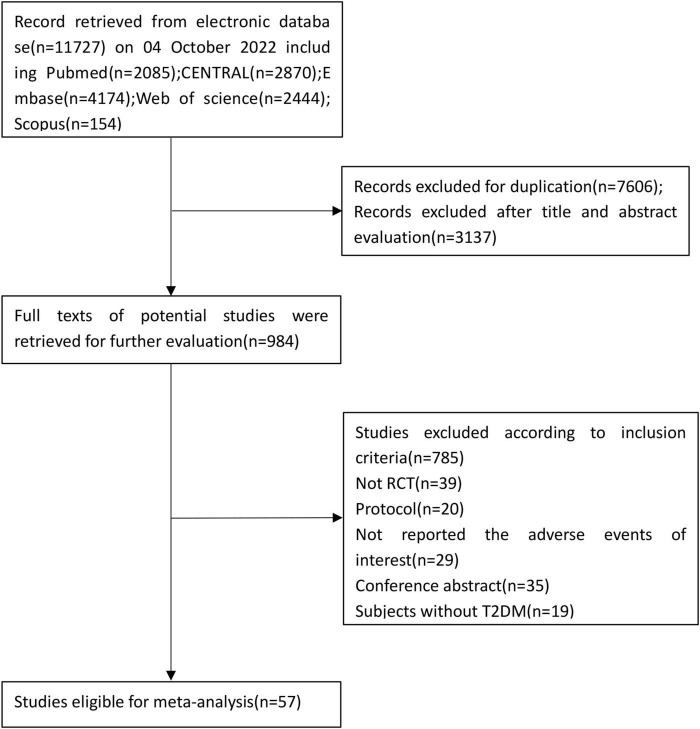
Flowchart showing identification of the included studies.

Figure 1 shows the result of our literature retrieval; in brief, 11,727 citations were initially screened and 7,606 duplications were excluded. An additional 3,137 articles were excluded based on their titles and abstracts. The remaining 984 citations were evaluated by inclusion criteria, further removing 927 citations. At last, there were 55 papers (57 studies) involving 68,622 patients that met the inclusion criteria at last (8–62). In the paper reported by Barnett (20), patients were recruited and randomized by the stage of chronic kidney disease, and the incident of hypovolemia was presented separately, thus each of them was considered as a separate study in this meta-analysis. Twelve RCTs (*n* = 11,576) evaluated canagliflozin, 22 RCTs (*n* = 36,508) evaluated dapagliflozin, 13 RCTs (*n* = 17,284) evaluated empagliflozin, four RCTs (*n* = 970) evaluated luseogliflozin, two RCTs (*n* = 310) evaluated ipragliflozin, two RCTs (*n* = 440) evaluated tofogliflozin, and the remaining one RCT (*n* = 312) evaluated bexagliflozin. The last one RCTs (*n* = 1,222) evaluated sotagliflozin. Among those 57 studies 51 studies compared SGLT2 inhibitors with a placebo, and another six studies compared SGLT2 inhibitors with other antidiabetic drugs. All eligible studies were randomized and double-blind design, and the publication year of studies ranged from 2009 to 2022, The follow-up duration of studies varies from 12 weeks to 208 weeks. The overview of the characteristics of included studies is presented in Table 1.

**TABLE 1:** Characteristics of the randomized controlled studies (RCTs) included in the meta-analysis.

Author	Trial identifier	Study duration	Intervention	control	Patients (n)		Age (years)		Duration of T2DM (years)		HbA1c (%)		Case of hypovolemia	
					Intervention	Control	Intervention	control	Intervention	control	Intervention	control	Intervention	control
Strojek et al. (10)	NCT00263276	12weeks	DAPA 2.5mg, 5mg, 10mg, 20mg, 50mg	PLA	59, 58, 47, 59, 56	54	55 ± 11, 55 ± 12, 54 ± 9, 55 ± 10, 53 ± 10	53 ± 11	NR	NR	7.6 ± 0.7, 8.0 ± 0.9, 8.0 ± 0.8, 7.7 ± 0.9, 7.8 ± 1.0	7.9 ± 0.9	0, 0, 0, 0, 2	1
Nauck et al. (11)	NCT00528879	24weeks	DAPA 2.5mg, 5mg, 10mg	PLA	137, 137, 135	137	55 ± 9.3, 54.3 ± 9.4, 52.7 ± 9.9	53.7 ± 10.3	6 ± 6.2, 6.4 ± 5.8, 6.1 ± 5.4	5.8 ± 5.1	7.99 ± 0.9, 8.17 ± 0.96, 7.92 ± 0.82	8.11 ± 0.96	0, 2, 0	1
Bailey et al. (12)	NCT00680745	24weeks	DAPA 2.5mg, 5mg, 10mg	PLA	154, 142, 151	145	59.9 ± 10.14, 60.2 ± 9.73, 58.9 ± 8.32	60.3 ± 10.16	7.7 ± 6.0, 7.4 ± 5.7, 7.2 ± 5.5	7.4 ± 5.7	8.11 ± 0.75, 8.12 ± 0.78, 8.07 ± 0.79	8.15 ± 0.74	1, 0, 1	0
Wilding et al. (13)	NCT00660907	52weeks	DAPA 10mg	GLIP 20mg	406	408	58 ± 9	59 ± 10	6 ± 5	7 ± 6	7.7 ± 0.9	7.7 ± 0.9	6	3
Cefalu et al. (14)	NR	24weeks	DAPA 1mg, 2.5mg, 5mg	PLA	72, 74, 68	68	53.7 ± 9.04, 53.5 ± 10.61, 51.3 ± 11.51	53.5 ± 11.08	1.6 ± 2.55, 1.5 ± 2.19, 1.4 ± 3.24	1.1 ± 1.95	7.8 ± 0.98, 8.1 ± 1.07, 7.9 ± 1.03	7.8 ± 1.12	0, 0, 1	0
Wilding et al. (15)	NCT00673231	24weeks	DAPA 2.5mg, 5mg, 10mg	PLA	202, 211, 194	193	59.8 ± 7.6, 59.3 ± 7.9, 59.3 ± 8.8	58.8 ± 8.6	13.6 ± 6.6, 13.1 ± 7.8, 14.2 ± 7.3	13.5 ± 7.3	8.46 ± 0.78, 8.62 ± 0.89, 8.57 ± 0.82	8.47 ± 0.77	5, 5, 4	2
Lavalle-Gonzalez et al. (16)	NCT00968812	52weeks	CANA 100mg, 300mg	GLIM 6/8mg	483, 485	482	56.4 ± 9.5, 55.8 ± 9.2	56.3 ± 9.0	6.5 ± 5.5, 6.7 ± 5.5	6.6 ± 5.0	7.8 ± 0.8, 7.8 ± 0.8	7.8 ± 0.8	4, 3	3
Schernthaner et al. (17)	NCT01106625	52weeks	CANA 100mg, 300mg	PLA	157, 156	156	57.4 ± 10.5, 56.1 ± 8.9	56.8 ± 8.3	9.0 ± 5.7, 9.4 ± 6.4	10.3 ± 6.7	8.1 ± 0.9, 8.1 ± 0.9	8.1 ± 0.9	1, 6	3
Stenlof et al. (18)	NCT01106677	52weeks	CANA 100mg, 300mg	SITA 100mg	368, 367	366	55.5 ± 9.4, 55.3 ± 9.2	55.5 ± 9.6	6.7 ± 5.4, 7.1 ± 5.4	6.8 ± 5.2	7.9 ± 0.9, 7.9 ± 0.9	7.9 ± 0.9	0, 0, 1	1
Bode et al. (19)	NCT01137812	52weeks	CANA 300mg	SITA 100mg	377	378	56.6 ± 9.6	56.7 ± 9.3	9.4 ± 6.1	9.7 ± 6.3	8.1 ± 0.9	8.1 ± 0.9	0	1
Barnett et al. (20)	NCT01081834	26weeks	CANA 100mg, 300mg	PLA	195, 197	192	55.1 ± 10.8, 55.3 ± 10.2	55.7 ± 10.9	4.5 ± 4.4, 4.3 ± 4.7	4.2 ± 4.1	8.1 ± 1.0, 8.0 ± 1.0	8.0 ± 1.0	0, 2	0
Bolinder et al. (21)	NCT01106651	26weeks	CANA 100mg, 300mg	PLA	241, 236	237	64.3 ± 6.5, 63.4 ± 6.0	63.2 ± 6.2	12.3 ± 7.8, 11.3 ± 7.2	11.4 ± 7.3	7.8 ± 0.8, 7.7 ± 0.8	7.8 ± 0.8	2, 1	0
Forst et al. (22)	NCT01164501	52weeks	EMPA 10mg, 25mg	PLA	98, 97	95	63.2 ± 8.5, 62 ± 8.4	62.6 ± 8.1	NR	NR	8.02 ± 0.84, 7.96 ± 0.73	8.09 ± 0.8	1, 0	1
Jabbour et al. (23)	NCT00855166	24weeks	DAPA 10mg	PLA	89	91	60.6 ± 8.2	60.8 ± 6.9	6.0 ± 4.5	5.5 ± 5.3	7.19 ± 0.44	7.16 ± 0.53	1	0
					Interven-tion	Control	Interven-tion	control	Intervention	control	Interven-tion	control	Interven-tion	control
Kadowaki et al. (24)	NCT01106690	52weeks	CANA 100mg, 300mg	PLA	113, 114	115	56.7 ± 10.4, 57 ± 10.2	58.3 ± 9.6	10.5 ± 6.6, 11 ± 7.6	10.1 ± 6.6	8.0 ± 0.9, 7.9 ± 0.9	8.0 ± 1.0	9, 5	4
Kohan et al. (25)	NCT00984867	24weeks	DAPA 10mg	PLA	223	224	54.8 ± 10.4	55.0 ± 10.2	5.70 ± 4.87	5.64 ± 5.4	7.9 ± 0.8	8.0 ± 0.8	3	2
Leiter et al. (26)	NCT01193218	12weeks	EMPA 5mg, 10mg, 25mg, 50mg	PLA	110, 109, 109, 110	109	57.3 ± 11.2, 57.9 ± 9.4, 57.2 ± 9.7, 56.6 ± 10.3	58.7 ± 8.7	NR	NR	7.92 ± 0.70, 7.93 ± 0.71, 7.93 ± 0.78, 8.02 ± 0.65	7.94 ± 0.74	0, 1, 1, 0	0
Seino et al. (27)	NCT00663260	104weeks	DAPA 5mg, 10mg	PLA	83, 85	84	66 ± 8.9, 68 ± 7.7	67 ± 8.6	16.9 ± 9.0, 18.2 ± 10.1	15.7 ± 9.5	8.30 ± 1.04, 8.22 ± 0.98	8.53 ± 1.28	8, 11	5
Seino et al. (28)	NCT01042977	24weeks	DAPA 10mg	PLA	480	482	63.9 ± 7.6	63.6 ± 7.0	13.5 ± 8.2	13.0 ± 8.4	8.0 ± 0.8	8.1 ± 0.8	7	13
Inagaki et al. (29)	JapicCTI-090908	12weeks	LUSE 0.5mg, 2.5mg, 5mg	PLA	60, 61, 61	54	55.2 ± 10.1, 58.3 ± 9.4, 56.8 ± 9.3	57.6 ± 11.0	4.90 ± 4.49, 6.15 ± 6.50, 5.77 ± 5.55	7.30 ± 6.43	8.16 ± 0.93, 8.07 ± 0.90, 8.16 ± 0.96	7.88 ± 0.72	1, 1, 0	0
Kaku et al. (30)	Japic CTI-101191	12weeks	LUSE 1mg, 2.5mg, 5mg, 10mg	PLA	55, 56, 54, 58	57	58.5 ± 9.1, 57.4 ± 9.3, 57.3 ± 11.4, 59.6 ± 7.8	57.1 ± 10.0	4.7 ± 4.1, 4.6 ± 4.4, 4.5 ± 4.2, 6.2 ± 5.4	5.1 ± 4.6	7.77 ± 0.79, 8.05 ± 0.75, 7.86 ± 0.69, 7.95 ± 0.67	7.92 ± 0.84	0, 2, 1, 3	0
Yale et al. (31)	NCT01413204	24weeks	CANA 100mg, 200mg	PLA	90, 88	93	58.4 ± 10.4, 57.4 ± 11.1	58.2 ± 11.0	4.72 ± 4.59, 5.88 ± 5.93	5.63 ± 5.76	7.98 ± 0.73, 8.04 ± 0.77	8.04 ± 0.70	0, 2	0
Ridderstrale et al. (32)	JapicCTI-101349	24weeks	TOFO 10mg, 20mg, 40mg	PLA	57, 58, 58	56	58.6 ± 9.8, 56.6 ± 10.2, 57.0 ± 9.1	56.8 ± 9.9	6.3 ± 7.1, 6.4 ± 5.1, 6.7 ± 5.5	6.0 ± 6.1	8.45 ± 0.75, 8.34 ± 0.81, 8.37 ± 0.77	8.41 ± 0.78	0, 0, 1	0
Cefalu et al. (33)	NCT01064414	26weeks	CANA 100mg, 300mg	PLA	90, 89	90	69.5 ± 8.2, 67.9 ± 8.2	68.2 ± 8.4	15.6 ± 7.4, 17.0 ± 7.8	16.4 ± 10.1	7.9 ± 0.9, 8.0 ± 0.8	8.0 ± 0.9	0, 1	0
Ji et al. (34)	NCT01167881	208weeks	EMPA 25mg	GLIM 1-4mg	765	780	56.2 ± 10.3	55.7 ± 10.4	NR	NR	7.92 ± 0.81	7.92 ± 0.86	20	15
Kovacs et al. (35)	NCT01031680	52weeks	DAPA 10mg	PLA	455	459	62.8 ± 7.0	63.0 ± 7.7	12.6 ± 8.7	12.3 ± 8.2	8.18 ± 0.84	8.08 ± 0.80	13	2
Merker et al. (36)	NCT01381900	18weeks	CANA 100mg, 300mg	PLA	223, 227	226	56.5 ± 8.3, 56.4 ± 9.2	55.8 ± 9.4	6.8 ± 4.5, 6.9 ± 4.9	6.4 ± 4.6	8.0 ± 0.9, 8.0 ± 0.9	7.9 ± 0.9	0, 1	0
Roden et al. (37)	NCT01210001	24weeks	EMPA 10mg, 25mg	PLA	165, 168	165	54.7 ± 9.9, 54.2 ± 8.9	54.6 ± 10.5	NR	NR	8.07 ± 0.89, 8.06 ± 0.82	8.16 ± 0.92	0, 2	0
Ross et al. (38)	NCT01159600	24weeks	EMPA 10mg, 25mg	PLA	217, 213	207	55.5 ± 9.9, 55.6 ± 10.2	56.0 ± 9.7	NR	NR	7.9 ± 0.8, 7.9 ± 0.9	7.9 ± 0.9	2, 1	0
Seino et al. (39)	NCT01289990	24weeks	EMPA 10mg, 25mg	PLA	224, 224	228	56.2 ± 11.6, 53.8 ± 11.6	54.9 ± 10.9	NR	NR	7.87 ± 0.88, 7.86 ± 0.85	7.91 ± 0.78	6, 2	1
Zinman et al. (40)	NR	16weeks	EMPA 12.5mg bid, 25mg, 5mg bid, 10mg	PLA	215, 214, 215, 214	107	57.6 ± 9.9, 58.2 ± 10.2, 58.8 ± 9.8, 58.5 ± 10.8	57.9 ± 11.2	NR	NR	7.78 ± 0.79, 7.73 ± 0.79, 7.79 ± 0.88, 7.84 ± 0.75	7.69 ± 0.72	1, 0, 0, 2	0
Matthaei et al. (41)	JapicCTI-111507	24weeks	LUSE 2.5mg	PLA	150	71	61.2 ± 8.4	59.9 ± 10.5	7.4 ± 5.6	7.9 ± 6.6	8.07 ± 0.85	8.01 ± 0.73	1	0
Tikkanen et al. (42)	NCT01131676	148.8we-eks	EMPA 10mg, 25mg	PLA	2345, 2342	2333	63.0 ± 8.6, 63.2 ± 8.6	63.2 ± 8.8	NR	NR	8.07 ± 0.86, 8.06 ± 0.84	8.08 ± 0.84	115, 124	115
Bailey (43)	NCT01392677	24weeks	DAPA 10mg	PLA	109	109	61.1 ± 9.7	60.9 ± 9.2	9.3 ± 6.5	9.6 ± 6.2	8.08 ± 0.91	8.24 ± 0.87	1	0
Ishihara et al. (44)	NCT01370005	12weeks	EMPA 10mg, 25mg	PLA	276, 276	271	60.6 ± 8.5, 59.9 ± 9.7	60.3 ± 8.8	NR	NR	7.87 ± 0.77, 7.92 ± 0.72	7.90 ± 0.72	0, 0	1
Rodbard et al. (45)	NCT00528372	102weeks	DAPA 2.5mg, 5mg, 10mg	PLA	65, 64, 70	75	53.0 ± 11.7, 52.6 ± 10.9, 50.6 ± 10.0	52.7 ± 10.3	2.1 ± 3.2, 1.0 ± 1.6, 2.3 ± 3.7	2.1 ± 3.1	7.92 ± 0.9, 7.86 ± 0.94, 8.01 ± 0.96	7.84 ± 0.87	0, 0, 1	1
Weber et al. (46)	NCT02175784	16weeks	IPRA 50mg	PLA	175	87	58.7 ± 11.1	59.2 ± 9.3	12.59 ± 7.79	14.28 ± 8.54	8.67 ± 0.77	8.62 ± 0.86	4	1
Wan Seman et al. (47)	NR	26weeks	CANA 100mg, 300mg	PLA	107	106	57.4 ± 9.3	57.5 ± 10.1	9.8 ± 5.4	10.1 ± 5.9	8.5 ± 0.9	8.4 ± 0.8	1	2
Weber et al. (48)	NCT01195662	12weeks	DAPA 10mg	PLA	225	224	NR	NR	7⋅7 ± 5⋅9	7⋅3 ± 5⋅0	8.1 ± 0.9	8.0 ± 1.0	1	0
Fioretto et al. (49)	NR	12weeks	DAPA 10mg	SU	58	52	53 ± 9.1	56 ± 9.1	5.0(3.0, 9.0)*	6.0(3.0, 10.3)*	7.7(7.08, 8.43)*	7.6 (6.9, 8.1)*	11	5
Seino et al. (50)	NCT01137474	12weeks	DAPA 10mg	PLA	302	311	55.6 ± 8.4	56.2 ± 8.9	8.2 ± 6.4	7.6 ± 6.2	8.1 ± 1.0	8.0 ± 0.9	1	0
Terauchi et al. (51)	NCT02413398	24weeks	DAPA 10mg	PLA	160	161	NR	NR	14.3 ± 8.1	14.5 ± 8.3	8.33 ± 1.08	8.03 ± 1.08	3	0
Yang et al. (52)	JapicCTI- 142582	16weeks	LUSE 2.5mg	PLA	159	74	57.4 ± 10.3	57.1 ± 10.9	11.7 ± 7.6	12.1 ± 6.8	8.70 ± 0.83	8.84 ± 0.83	7	1
Allegretti et al. (53)	NCT02201004	16weeks	TOFO 20mg	PLA	141	70	59.1 ± 10.8	56.4 ± 10.0	15.02 ± 9.36	12.39 ± 7.34	8.53 ± 0.75	8.40 ± 0.65	11	2
Wiviott et al. (54)	NCT02096705	24weeks	DAPA 10mg	PLA	139	133	56.5 ± 8.4	58.6 ± 8.9	12.7 ± 7.2	12.2 ± 6.7	8.52 ± 0.76	8.58 ± 0.81	1	0
Mahaffey et al. (55)	NCT02836873	24weeks	BEXA 20mg	PLA	157	155	69.3 ± 8.36	69.9 ± 8.29	15.54 ± 9.198	16.28 ± 8.977	8.01 ± 0.786	7.95 ± 0.812	6	5
Pollock et al. (56)	NCT01730534	201.6we-eks	DAPA 10mg	PLA	8582	8578	63.9 ± 6.8	64 ± 6.8	NR	NR	NR	NR	213	207
Inoue et al. (57)	NCT02065791	26weeks	CANA 100mg	PLA	2202	2199	62.85 ± 8.95	63.15 ± 9.15	15.55 ± 8.65	16 ± 8.55	8.25 ± 1.3	8.3 ± 1.3	144	115
McMurray et al. (58)	NCT02547935	24weeks	DAPA 10mg	PLA	145	148	64.7 ± 8.6	64.7 ± 8.5	17.55 ± 7.7	17.71 ± 9.5	8.44 ± 1.0	8.57 ± 1.2	4	4
Packer et al. (59)	UMIN0000 18839	24weeks	IPRA 50mg	PLA	24	24	60.5 ± 9.8	60.8 ± 12.1	15.9 ± 7.7	19.1 ± 10.7	8.12 ± 0.93	8.30 ± 0.65	1	0
Lee et al. (60)	NCT03036124	72.8weeks	DAPA 10mg	PLA	2373	2371	66.2 ± 11.0	66.5 ± 10.8	NR	NR	NR	NR	2	5
Bhatt et al. (61)	NCT03057977	64weeks	EMPA 10mg	PLA	1863	1867	67.2 ± 10.8	66.5 ± 11.2	NR	NR	NR	NR	197	184
Solomon et al. (62)	NCT03485092	36 weeks	EMPA 10mg	PLA	52	53	68.7 ± 11.1	68.2 ± 11.7	9.7 ± 6.8	9.0 ± 6.2	7.2 ± 1.5	7.5 ± 1.6	29	31
Neal et al. (63)	NCT03521934	36weeks	SOTA 200mg	PLA	608	614	69(63–76)*	70(64–76)*	NR	NR	7.1(6.4–8.3)*	7.2(6.4–8.2)*	57	54
O’Meara et al. (64)	NCT03619213	110.4weeks	DAPA 10mg	PLA	3131	3132	71.8 ± 9.6	71.5 ± 9.5	NR	NR	NR	NR	42	32

Data are the number of patients (*n*)or mean (sd) unless stated otherwise; *Median (IQR). BMI, body mass index; HbA1c, glycated hemoglobin; IQR, interquartile range; s.d., standard deviation; DAPA, dapagliflozin; CANA, canagliflozin; IPRA, ipragliflozin; TOFO, tofogliflozin; EMPA, empagliflozin; BEXA, bexagliflozi; LUSE, luseogliflozin; SOTA, Sotagliflozin; PLA, placebo; GLIM, glimepiride; GLIP, glipizide; SITA, sitagliptin; SEMA, semaglutide; SU, sulphonylurea; NR, not report.

### Quality assessment

**FIGURE 2:**
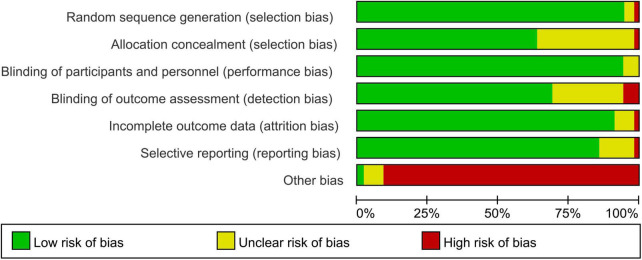
Risk of bias and methodologic quality of the randomized controlled trials. Green represents low risk of bias, red represents high risk of bias, and yellow indicates unclear risk.

We applied Cochrane risk-of-bias tool to assess the study quality. Detailed information about risk-of-bias is presented in Figure 2. Overall, taking the risk of sponsorship bias into consideration, all studies’ other sources of bias were assessed as high. With respect to methods of sequence generation, allocation concealment, and/or blinding of patients and personnel, 25 RCTs had not provided enough information to evaluate.

### Publication bias and sensitivity analysis

**FIGURE 3:**
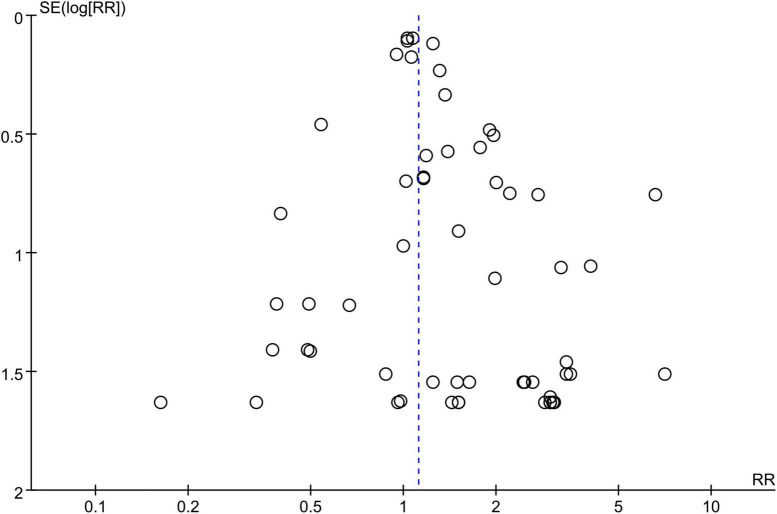
Funnel plot of publication bias for hypovolemia comparing the SGLT2 inhibitors group vs. the placebo group.

A symmetrical funnel plot of SGLT2 inhibitors vs. placebo for hypovolemia indicated no evidence of publication bias (Figure 3). Begg’s test signified there was no publication bias in the included studies (*z* = 0.06; *P* = 0.954). We performed the trim-and-fill method to detect and adjust for publication bias; after trim-and-fill, the pooled RR was 1.117 (95% CI: 1.025; 1.218), which was approximately equal to the original pooled RR (1.12). Collectively, in the result of the funnel plot, Begg’s test, and trim-and-fill method, there was no evidence of publication bias. The result of sensitivity analyses indicated that the combined RRs were all not statistically significant and were similar to one another, with a range from 1.14 (95% CI: 1.052; 1.253) to 1.20 (95% CI: 1.088; 1.326). This implied that the result of our meta-analysis was robust.

### Sodium–glucose cotransporter-2 inhibitors and hypovolemia

**FIGURE 4:**
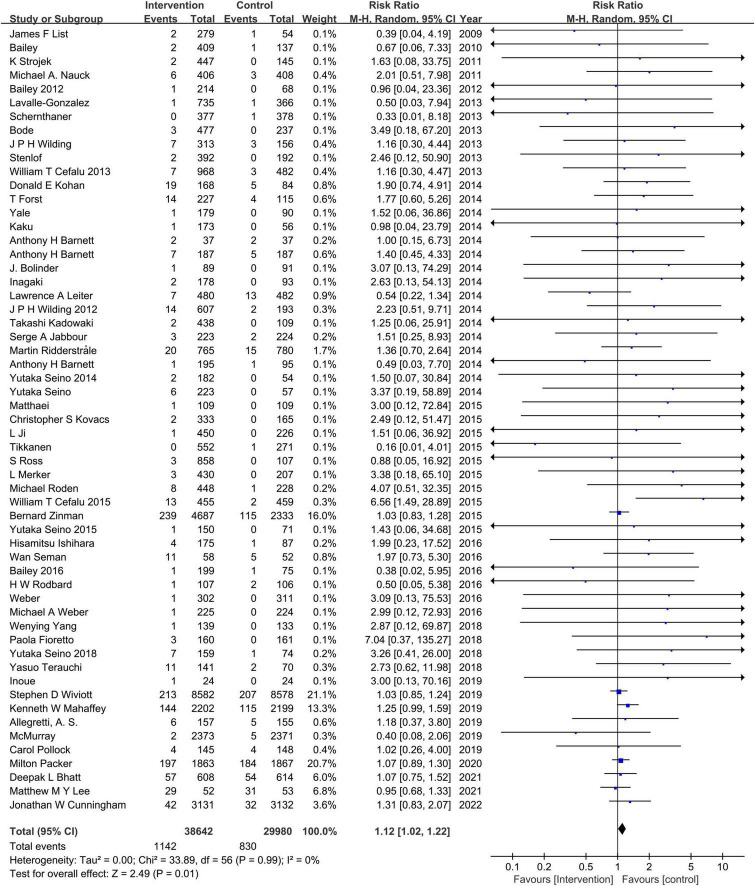
Comparisons of hypovolemia events between the SGLT2 inhibitors group vs. the placebo group.

**FIGURE 5:**
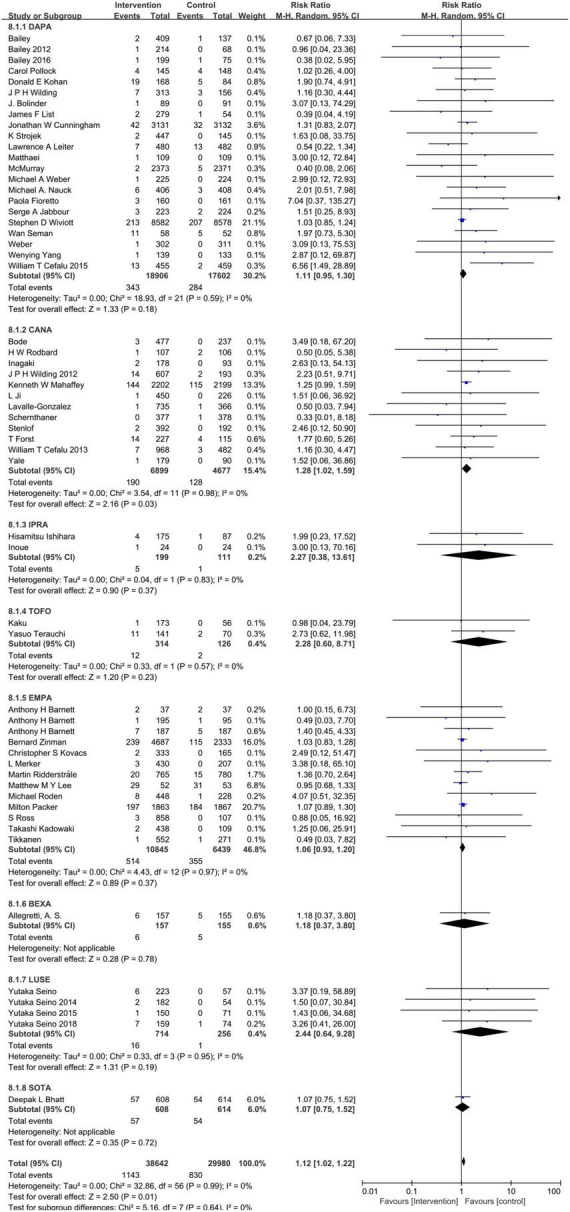
Subgroup analysis of the hypovolemia events between the SGLT2 inhibitors group vs. placebo group stratified by category of SGLT2 inhibitors. DAPA, dapagliflozin; CANA, canagliflozin; IPRA, ipragliflozin; TOFO, Tofogliflozin; EMPA, empagliflozin; BEXA, bexagliflozin; LUSE, luseogliflozin; SOTA, Sotagliflozin.

**FIGURE 6:**
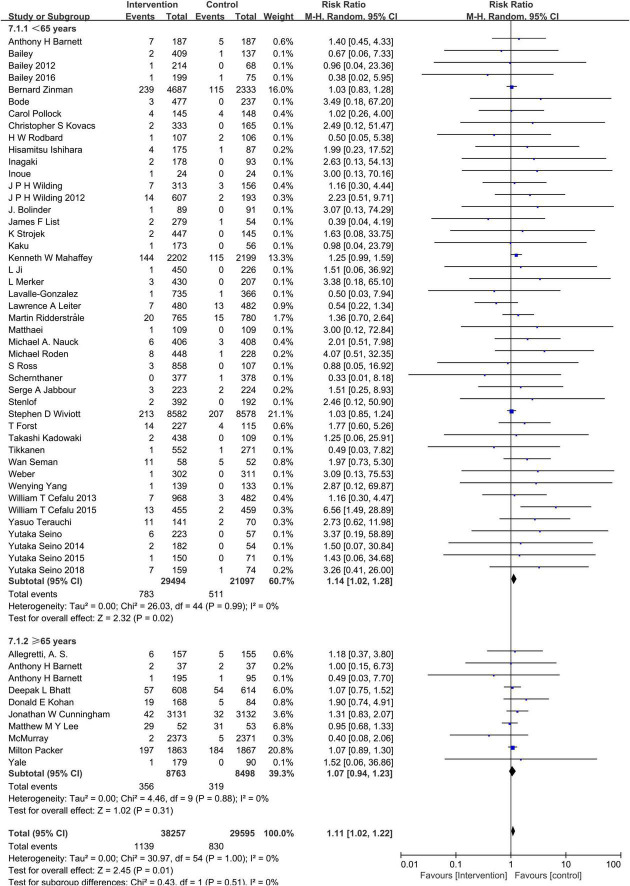
Subgroup analysis of the hypovolemia events between the SGLT2 inhibitors group vs. the placebo group stratified by age.

**FIGURE 7:**
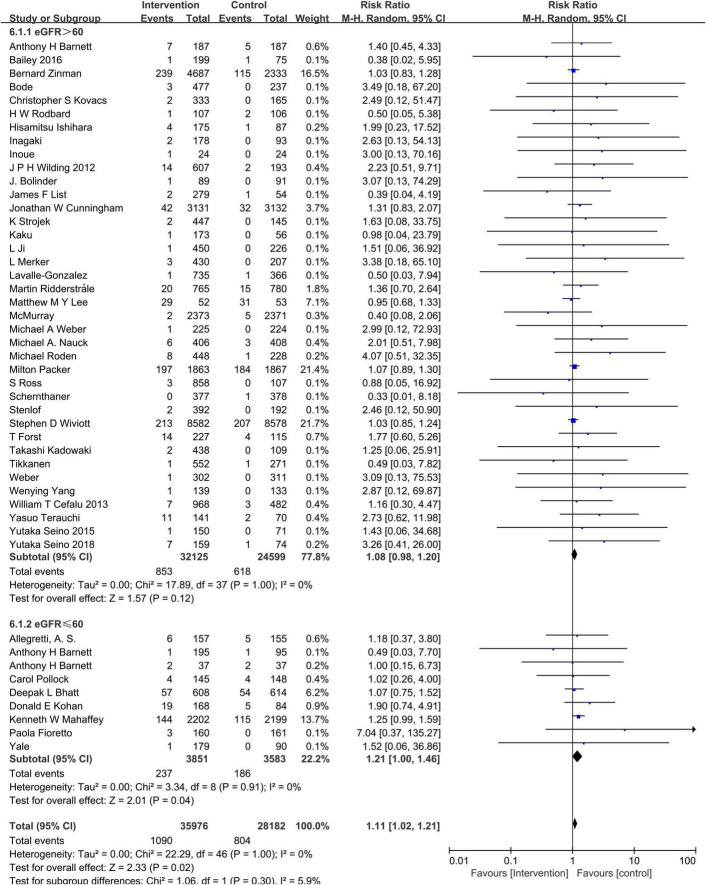
Subgroup analysis of the hypovolemia events between the SGLT2 inhibitors group vs. the placebo group stratified by eGFRs.

Compared to the placebo, the combined RR of SGLT2 inhibitors was 1.12 (95% CI:1.02–1.22). The heterogeneity, as assessed by *I*^2^, was 0% (*P* = 0.99), suggesting there was no significant heterogeneity between studies (Figure 4). Additional subgroup analyses were performed to assess the effect of the category of SGLT2 inhibitors, patients’ age, and baseline eGFR on the risk of hypovolemia. When stratified by category of SGLT2 inhibitors, the pooled RRs of sotagliflozin, luseogliflozin, Tofogliflozin, ipragliflozin, canagliflozin, bexagliflozin, dapagliflozin, and empagliflozin were 1.07 (95% CI:0.75–1.52), 2.44 (95% CI:0.64–9.28), 2.28 (95% CI:0.60–8.71), 2.27 (95% CI:0.38–13.61), 1.28 (95% CI:1.02–1.59), 1.18 (95% CI:0.37–3.80), 1.11 (95% CI:0.95–1.30), and 1.06 (95% CI:0.93–1.20), respectively (Figure 5). When the subgroup was analyzed by age, the pooled RR of patients with age ≥65 years and those with age < 65 years were (1.07; 95% CI: 0.94–1.23) and (1.14; 95% CI: 1.02–1.28), respectively (Figure 6). The pooled RR (1.21, 95% CI: 1.00–1.46) was slightly higher in the subgroup with a baseline eGFR less than or equal to 60 mL/min/1.73 m^2^ than in the subgroup with a baseline eGFR greater than 60 mL/min/1.73 m^2^ (RR, 1.08; 95% CI: 0.98–1.20), but there was no statistical significance for eGFR subgroup differences (*p* = 0.30) (Figure 7).

## Discussion

With 57 included RCTs involving 68,622 patients, this meta-analysis indicated that SGLT2 inhibitors increased the risk of hypovolemia, especially in patients with older age and lower eGFR. Both EMPA-REG OUTCOME trial and the CANVAS trial demonstrated that prescribing SGLT2 inhibitors reduces the risk of hospitalization for heart failure (40, 63). Researchers subsequently conducted a serial clinical trial to confirm this prevailing benefit. The recently finished DAPA-HF trial suggests that dapagliflozin decreases the risk of worsening heart failure or death from cardiovascular causes among those with or without T2DM (58). Although the 2020 CCS/CHFS heart failure guidelines recommended applicating SGLT2 inhibitors to heart failure patients with or without T2DM (64), the mechanisms underpinning the cardioprotective effects of SGLT2 inhibitors remain in debate (65). Natriuresis and osmotic diuresis of SGLT2 inhibitors was thought to play a crucial role in cardioprotective effects (66). Just like every coin has two sides, depending on the osmotic diuresis of SGLT2 inhibitors, it is plausible to consider the possibility of the increased risk of volume depletion with caution. Although individual large multicentral RCTs reported the frequency of adverse events related to volume depletion did not differ between SGLT2 inhibitors and control groups (59, 62). Because of the low statistical power of individual studies and the heterogeneity of the population included in studies (e.g., with or without diabetes), the conclusion remained to be discussed. There is also a lot of meta-analysis evaluating the efficacy and safety of SGLT2 inhibitors, the majority of them focuses on analyzing the risk of hypoglycemia, and genital and urinary tract infections (67). Some of them threw light on the osmotic diuresis-related adverse event, with the result of there being no evidence of SGLT2 inhibitors increasing the risk of hypovolemia (68). This conclusion is not consistent with our meta-analysis. We thought there are three explanations for this inconsistency. First, in contrast to the earlier analysis, our meta-analysis covered a wider spectrum of the category of SGLT2 inhibitors. Second, adopting different analysis methods could impact the result. At last, the differences in included RCTs could also contribute to the inconsistency.

Our meta-analysis also investigated the category of SGLT2 inhibitors, patients’ age, and baseline eGFR impact on the risk of hypovolemia associated with SGLT2 inhibitors treatment. When stratified by category of SGLT2 inhibitors, luseogliflozin, Tofogliflozin and ipragliflozin had RRs greater than 2. It should be noted that they exist a broad 95% CIs as well, which implies the imprecision of statistics. It should be cautious to interpret this point. Canagliflozin showed an increased risk of hypovolemia compared with dapagliflozin or empagliflozin. This might be explained by canagliflozin having the lowest SGLT2/SGLT1 affinity ratio and empagliflozin the highest (69). People aged ≥65 years and with baseline eGFR <60 mL/min/1.73 m^2^ were more predisposed to hypovolemia; the deteriorative kidney function could be responsible for the conclusion.

**FIGURE 8:**
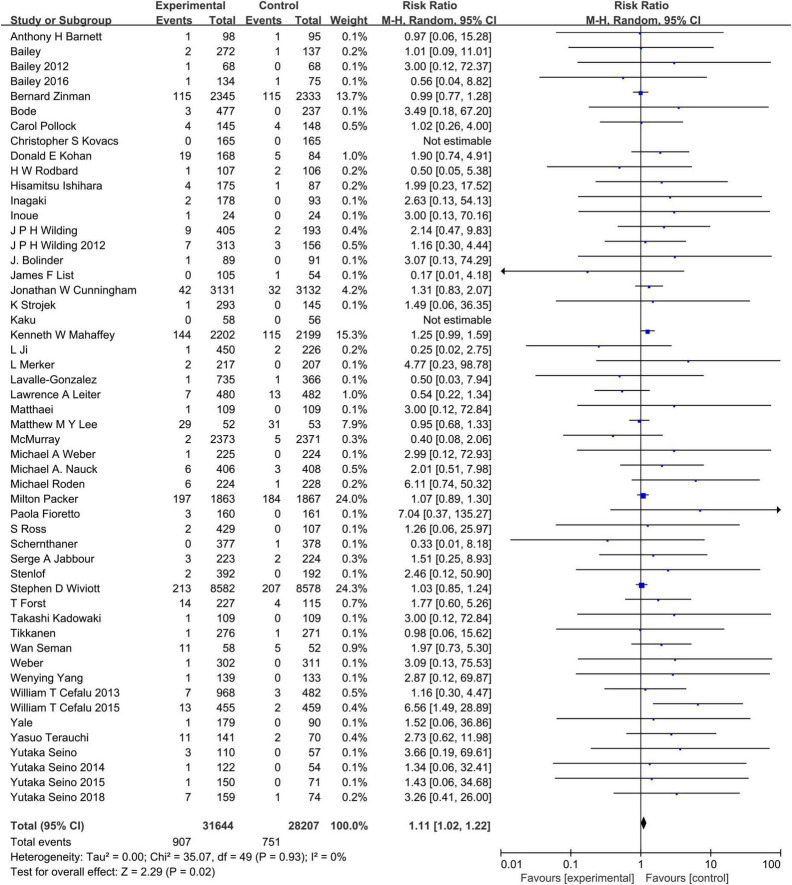
Subgroup analysis of the hypovolemia events between the SGLT2 inhibitors group vs. the placebo group stratified by FDA approval dosage.

We further noticed that our meta-analysis included several trials that tested high doses of SGLT2 inhibitors, such as Dapagliflozin 50 mg, Empagliflozin 12.5 mg, Empagliflozin 25 mg, and so on. With the concerns of whether the result may be influenced by these dosages, we performed an additional subgroup analysis depending on the dosage of SGLT2 with U.S. Food and Drug Administration (FDA) approval. As presented in Figure 8, even though excluded the study arms of dosages that were without FDA approval, the combined RR of SGLT2 was 1.11(95% CI:1.02–1.22), which means SGLT2 inhibitors increase the risk of hypovolemia. So, we conclude that the result could not be influenced by high doses of SGLT2 inhibitors.

**FIGURE 9:**
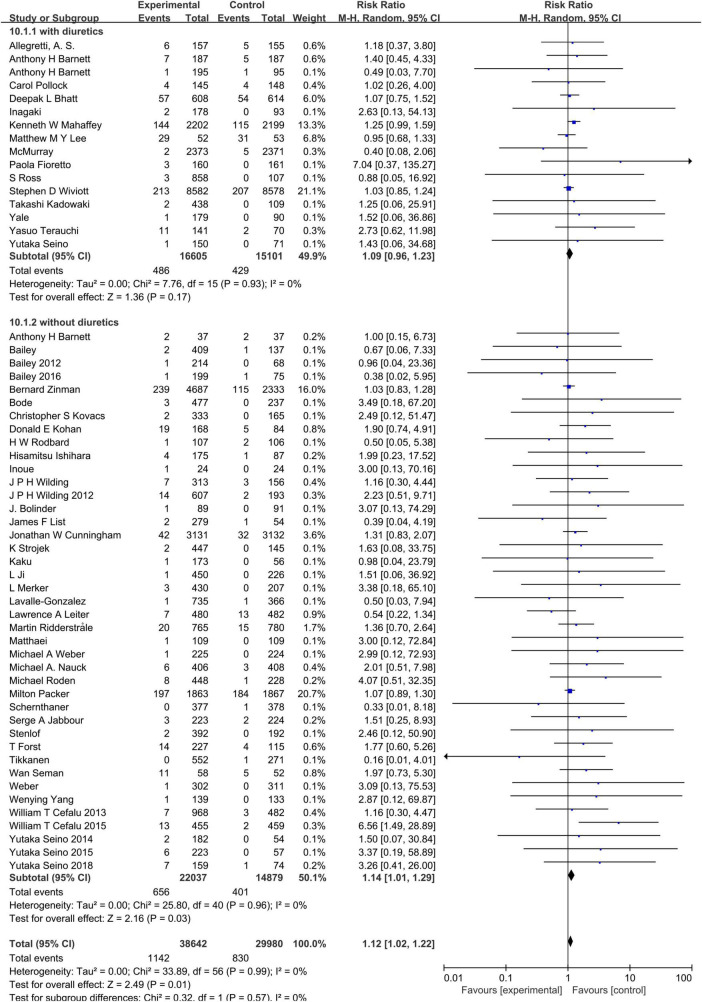
Subgroup analysis of the hypovolemia events between the SGLT2 inhibitors group vs. the placebo group stratified by diuretics treatment.

Because diuretics might augment the effect of SGLT2 inhibitors, with increasing risk of hypovolemia. The issue of whether the increased risk of hypovolemia associated with SGLT2 inhibitors ascribes to the diuretic treatment should be examined. Among the included 57 studies, there were 15 citations (20, 24, 29, 31, 38, 39, 49, 51, 53–55, 60, 61) providing the information on SGLT2 inhibitors add-on to the background diuretic treatment. Figure 9 shows that the pooled RR of interest in patients not treated without a diuretic at baseline is 1.14 (95% CI: 1.01–1.29) vs. 1.09 (95%CI: 0.96–1.23) in patients treated with a diuretic and the *P* for interaction is 0.57, which implied the difference of subgroup did not be attributed to diuretic treatment.

Collectively, the result of our meta-analysis, that SGLT2 inhibitors increased the risk of hypovolemia, was robust.

This meta-analysis also had some limitations. First, the assessment of hypovolemia was based on a predefined list of preferred terms. This strategy may underestimate the risk of hypovolemia associated with SGLT2 inhibitors. Second, the pooled data was basically drawn from 22 RCTs evaluating dapagliflozin,12 RCTs with canagliflozin, and 13 RCTs with empagliflozin, it should be cautious to extrapolate the conclusion to another category of SGLT2 inhibitors. Finally, we were unable to further stratify the concomitant drug, due to the paucity of data provided by eligible RCTs.

In conclusion, our present meta-analysis indicated that increased risk of hypovolemia associated with SGLT2 inhibitors in T2DM patients. The incidence of hypovolemia is different among the category of SGLT2 inhibitors and may keep rising with the age, and degeneration of renal function. To achieve an optimal fluid volume in patients with heart failure is an exquisite skill, hypovolemia in HF patients may be detrimental (70). Our meta-analysis unveils the increasing risk of hypovolemia associated with SGLT2 inhibitors, if ignoring this risk, hypovolemia resulting in hypotension and renal hypoperfusion may overwhelm the benefit of SGLT2 inhibitors treatment. It is necessary to pay attention for the risk of hypovolemia associated with SGLT2 inhibitors under the context of some heart failure guidelines recommending prescribe SGLT2 inhibitors for heart failure patients with or without T2DM. The interrelation between SGLT2 inhibitors and hypovolemia and the underlying mediating mechanisms of cardio-protection deserves to be further investigated. It is important to take the advantage of the cardio-protection effect of SGLT2 inhibitors to the maximum extent and avoid the risk of hypovolemia associated with SGLT2 inhibitors to the utmost. It is also intriguing to conduct large-scale, multicenter, double-blind, head-to-head RCTs to compare the efficacy and safety of SGLT2 inhibitors with diuretics.

## Conclusion

In summary, the present meta-analysis indicated that SGLT2 inhibitors increased the risk of hypovolemia in patients with T2DM. The incidence of hypovolemia may increase with the decreasing of eGFR and growth of age. It is important to pay attention to the potential adverse event, except the common adverse event such as hypoglycemia, and genitourinary infection. Our meta-analysis indicates that there is an increase in hypovolemia associated with SGLT2 inhibitors treatment. It is necessary to be concerned about the risk of hypovolemia associated with SGLT2 inhibitors, especially in older individuals and those with moderate renal impairment.

## Data availability statement

The original contributions presented in this study are included in the article/Supplementary material, further inquiries can be directed to the corresponding author/s.

## Author contributions

XR was involved in designing the study, literature retrieval, quality assessment, and manuscript writing. YZ, BW, XL, and QG contributed to the extract data. KL provided suggestions on carrying out the study. XC was involved in manuscript preparation. All authors contributed to the study’s conception and design.
