# Supplementary material for: Risk of hypovolemia associated with sodium–glucose cotransporter-2 inhibitors treatment: A meta-analysis of randomized controlled trials
Source: Front Cardiovasc Med. 2022 Nov 14;9:973129. doi: 10.3389/fcvm.2022.973129 (PMC9701837; doi:10.3389/fcvm.2022.973129)
Supplement: Supplementary file 1 [file Table_1.DOCX]

| Author | publication | Trial Identifier | duration | Intervention | control | Patients (n) | | Age (years) | | Duration of T2DM (years) | | HbA1c (%) | | Case of hypovolemia | |
| --- | --- | --- | --- | --- | --- | --- | --- | --- | --- | --- | --- | --- | --- | --- | --- |
|  | Year of |  | Study |  |  | Intervention | controls | Intervention | controls | Intervention | controls | Intervention | controls | Intervention | controls |
| James F List(10) | 2009 | NCT00263276 | 12weeks | DAPA 2.5mg,5mg,10mg 20mg,50mg | PLA | 59,58,47,59,56 | 54 | 55±11,55±12,54±9, 55±10,53±10 | 53±11 | NR | NR | 7.6±0.7,8.0±0.9, 8.0±0.8,7.7±0.9, 7.8±1.0 | 7.9±0.9 | 0,0,0,0,2 | 1 |
| Bailey et al(11) | 2010 | NCT00528879 | 24weeks | DAPA 2.5mg,5mg,10mg | PLA | 137,137,135 | 137 | 55±9.3, 54.3±9.4, 52.7±9.9 | 53.7±10.3 | 6±6.2, 6.4±5.8, 6.1±5.4 | 5.8±5.1 | 7.99±0.9, 8.17±0.96, 7.92±0.82 | 8.11±0.96 | 0,2,0 | 1 |
| K Strojek(12) | 2011 | NCT00680745 | 24weeks | DAPA 2.5mg,5mg,10mg | PLA | 154,142,151 | 145 | 59.9±10.14,60.2±9.73, 58.9±8.32 | 60.3±10.16 | 7.7±6.0,7.4±5.7, 7.2±5.5 | 7.4±5.7 | 8.11±0.75,8.12±0.78, 8.07±0.79 | 8.15±0.74 | 1,0,1 | 0 |
| Michael A. Nauck(13) | 2011 | NCT00660907 | 52weeks | DAPA 10mg | GLIP 20mg | 406 | 408 | 58±9 | 59±10 | 6±5 | 7±6 | 7.7±0.9 | 7.7±0.9 | 6 | 3 |
| Bailey et al(14) | 2012 | NR | 24weeks | DAPA 1mg,2.5mg 5mg | PLA | 72,74,68 | 68 | 53.7±9.04,53.5±10.61, 51.3±11.51 | 53.5±11.08 | 1.6±2.55,1.5±2.19, 1.4±3.24 | 1.1±1.95 | 7.8±0.98,8.1±1.07, 7.9±1.03 | 7.8±1.12 | 0,0,1 | 0 |
| J P H Wilding(15) | 2012 | NCT00673231 | 24weeks | DAPA 2.5mg,5mg,10mg | PLA | 202,211,194 | 193 | 59.8±7.6,59.3±7.9, 59.3±8.8 | 58.8±8.6 | 13.6±6.6,13.1±7.8, 14.2±7.3 | 13.5±7.3 | 8.46±0.78,8.62±0.89, 8.57±0.82 | 8.47±0.77 | 5,5,4 | 2 |
| William T Cefalu(16) | 2013 | NCT00968812 | 52weeks | CANA 100mg,300mg | GLIM 6/8mg | 483,485 | 482 | 56.4±9.5,55.8±9.2 | 56.3±9.0 | 6.5±5.5,6.7±5.5 | 6.6±5.0 | 7.8±0.8, 7.8±0.8 | 7.8±0.8 | 4,3 | 3 |
| J P H Wilding(17) | 2013 | NCT01106625 | 52weeks | CANA 100mg,300mg | PLA | 157,156 | 156 | 57.4±10.5,56.1±8.9 | 56.8±8.3 | 9.0±5.7,9.4±6.4 | 10.3±6.7 | 8.1±0.9,8.1±0.9 | 8.1±0.9 | 1,6 | 3 |
| Lavalle-Gonzalez et al(18) | 2013 | NCT01106677 | 52weeks | CANA 100mg,300mg | SITA 100mg | 368,367 | 366 | 55.5±9.4, 55.3±9.2 | 55.5±9.6 | 6.7±5.4, 7.1±5.4 | 6.8±5.2 | 7.9±0.9, 7.9±0.9 | 7.9±0.9 | 0,0,1 | 1 |
| Schernthaner et al(19) | 2013 | NCT01137812 | 52weeks | CANA 300mg | SITA 100mg | 377 | 378 | 56.6±9.6 | 56.7±9.3 | 9.4±6.1 | 9.7±6.3 | 8.1±0.9 | 8.1±0.9 | 0 | 1 |
| Stenlof et al(20) | 2013 | NCT01081834 | 26weeks | CANA 100mg,300mg | PLA | 195,197 | 192 | 55.1±10.8, 55.3±10.2 | 55.7±10.9 | 4.5±4.4, 4.3±4.7 | 4.2±4.1 | 8.1±1.0, 8.0±1.0 | 8.0±1.0 | 0,2 | 0 |
| Bode et al(21) | 2013 | NCT01106651 | 26weeks | CANA 100mg,300mg | PLA | 241,236 | 237 | 64.3±6.5, 63.4±6.0 | 63.2±6.2 | 12.3±7.8, 11.3±7.2 | 11.4±7.3 | 7.8±0.8, 7.7±0.8 | 7.8±0.8 | 2,1 | 0 |
| Anthony H Barnett(22) | 2014 | NCT01164501 | 52weeks | EMPA 10mg,25mg | PLA | 98,97 | 95 | 63.2±8.5,62±8.4 | 62.6±8.1 | NR | NR | 8.02±0.84,7.96±0.73 | 8.09±0.8 | 1,0 | 1 |
| J. Bolinder(23) | 2014 | NCT00855166 | 24weeks | DAPA 10mg | PLA | 89 | 91 | 60.6±8.2 | 60.8±6.9 | 6.0±4.5 | 5.5±5.3 | 7.19±0.44 | 7.16±0.53 | 1 | 0 |
| T Forst(24) | 2014 | NCT01106690 | 52weeks | CANA 100mg,300mg | PLA | 113,114 | 115 | 56.7±10.4,57±10.2 | 58.3±9.6 | 10.5±6.6,11±7.6 | 10.1±6.6 | 8.0±0.9,7.9±0.9 | 8.0±1.0 | 9,5 | 4 |
| Serge A Jabbour(25) | 2014 | NCT00984867 | 24weeks | DAPA 10mg | PLA | 223 | 224 | 54.8±10.4 | 55.0±10.2 | 5.70±4.87 | 5.64±5.4 | 7.9±0.8 | 8.0±0.8 | 3 | 2 |
| Takashi Kadowaki(26) | 2014 | NCT01193218 | 12weeks | EMPA 5mg,10mg,25mg, 50mg | PLA | 110,109,109,110 | 109 | 57.3±11.2,57.9±9.4, 57.2±9.7,56.6±10.3 | 58.7±8.7 | NR | NR | 7.92±0.70,7.93±0.71, 7.93±0.78,8.02±0.65 | 7.94±0.74 | 0,1,1,0 | 0 |
| Donald E Kohan(27) | 2014 | NCT00663260 | 104weeks | DAPA 5mg,10mg | PLA | 83,85 | 84 | 66±8.9,68±7.7 | 67±8.6 | 16.9±9.0,18.2±10.1 | 15.7±9.5 | 8.30±1.04,8.22±0.98 | 8.53±1.28 | 8,11 | 5 |
| Lawrence A Leiter(28) | 2014 | NCT01042977 | 24weeks | DAPA 10mg | PLA | 480 | 482 | 63.9±7.6 | 63.6±7.0 | 13.5±8.2 | 13.0±8.4 | 8.0±0.8 | 8.1±0.8 | 7 | 13 |
| Yutaka Seino(29) | 2014 | JapicCTI-090908 | 12weeks | LUSE 0.5mg,2.5mg,5mg | PLA | 60,61,61 | 54 | 55.2±10.1,58.3±9.4 56.8±9.3 | 57.6±11.0 | 4.90±4.49,6.15±6.50, 5.77±5.55 | 7.30±6.43 | 8.16±0.93,8.07±0.90, 8.16±0.96 | 7.88±0.72 | 1,1,0 | 0 |
| Yutaka Seino(30) | 2014 | Japic CTI-101191 | 12weeks | LUSE 1mg,2.5mg,5mg, 10mg | PLA | 55,56,54,58 | 57 | 58.5±9.1,57.4±9.3 57.3±11.4,59.6±7.8 | 57.1±10.0 | 4.7±4.1,4.6±4.4, 4.5±4.2,6.2±5.4 | 5.1±4.6 | 7.77±0.79,8.05±0.75, 7.86±0.69,7.95±0.67 | 7.92±0.84 | 0,2,1,3 | 0 |
| Inagaki et al(31) | 2014 | NCT01413204 | 24weeks | CANA 100mg,200mg | PLA | 90,88 | 93 | 58.4±10.4, 57.4±11.1 | 58.2±11.0 | 4.72±4.59, 5.88±5.93 | 5.63±5.76 | 7.98±0.73, 8.04±0.77 | 8.04±0.70 | 0,2 | 0 |
| Kaku et al(32) | 2014 | JapicCTI-101349 | 24weeks | TOFO 10mg,20mg, 40mg | PLA | 57,58,58 | 56 | 58.6±9.8, 56.6±10.2, 57.0±9.1 | 56.8±9.9 | 6.3±7.1, 6.4±5.1, 6.7±5.5 | 6.0±6.1 | 8.45±0.75, 8.34±0.81, 8.37±0.77 | 8.41±0.78 | 0,0,1 | 0 |
| Yale et al(33) | 2014 | NCT01064414 | 26weeks | CANA 100mg,300mg | PLA | 90,89 | 90 | 69.5±8.2, 67.9±8.2 | 68.2±8.4 | 15.6±7.4, 17.0±7.8 | 16.4±10.1 | 7.9±0.9, 8.0±0.8 | 8.0±0.9 | 0,1 | 0 |
| Martin Ridderstråle(34) | 2014 | NCT01167881 | 208weeks | EMPA 25mg | GLIM 1-4mg | 765 | 780 | 56.2±10.3 | 55.7±10.4 | NR | NR | 7.92±0.81 | 7.92±0.86 | 20 | 15 |
| William T Cefalu(35) | 2015 | NCT01031680 | 52weeks | DAPA 10mg | PLA | 455 | 459 | 62.8±7.0 | 63.0±7.7 | 12.6±8.7 | 12.3±8.2 | 8.18±0.84 | 8.08±0.80 | 13 | 2 |
| L Ji(36) | 2015 | NCT01381900 | 18weeks | CANA 100mg,300mg | PLA | 223,227 | 226 | 56.5±8.3,56.4±9.2 | 55.8±9.4 | 6.8±4.5,6.9±4.9 | 6.4±4.6 | 8.0±0.9,8.0±0.9 | 7.9±0.9 | 0,1 | 0 |
| Christopher S Kovacs(37) | 2015 | NCT01210001 | 24weeks | EMPA 10mg,25mg | PLA | 165,168 | 165 | 54.7±9.9,54.2±8.9 | 54.6±10.5 | NR | NR | 8.07±0.89,8.06±0.82 | 8.16±0.92 | 0,2 | 0 |
| L Merker(38) | 2015 | NCT01159600 | 24weeks | EMPA 10mg,25mg | PLA | 217,213 | 207 | 55.5±9.9,55.6±10.2 | 56.0±9.7 | NR | NR | 7.9±0.8,7.9±0.9 | 7.9±0.9 | 2,1 | 0 |
| Michael Roden(39) | 2015 | NCT01289990 | 24weeks | EMPA 10mg,25mg | PLA | 224,224 | 228 | 56.2±11.6,53.8±11.6 | 54.9±10.9 | NR | NR | 7.87±0.88,7.86±0.85 | 7.91±0.78 | 6,2 | 1 |
| S Ross(40) | 2015 | NR | 16weeks | EMPA 12.5mg bid,25mg 5mg bid,10mg | PLA | 215,214,215,214 | 107 | 57.6±9.9,58.2±10.2 58.8±9.8,58.5±10.8 | 57.9±11.2 | NR | NR | 7.78±0.79,7.73±0.79 7.79±0.88,7.84±0.75 | 7.69±0.72 | 1,0,0,2 | 0 |
| Yutaka Seino(41) | 2015 | JapicCTI-111507 | 24weeks | LUSE 2.5mg | PLA | 150 | 71 | 61.2±8.4 | 59.9±10.5 | 7.4±5.6 | 7.9±6.6 | 8.07±0.85 | 8.01±0.73 | 1 | 0 |
| Bernard Zinman(42) | 2015 | NCT01131676 | 148.8weeks | EMPA 10mg,25mg | PLA | 2345 2342 | 2333 | 63.0±8.6,63.2±8.6 | 63.2±8.8 | NR | NR | 8.07±0.86,8.06±0.84 | 8.08±0.84 | 115,124 | 115 |
| Matthaei et al(43) | 2015 | NCT01392677 | 24weeks | DAPA 10mg | PLA | 109 | 109 | 61.1±9.7 | 60.9±9.2 | 9.3±6.5 | 9.6±6.2 | 8.08±0.91 | 8.24±0.87 | 1 | 0 |
| Tikkanen et al(44) | 2015 | NCT01370005 | 12weeks | EMPA 10mg,25mg | PLA | 276,276 | 271 | 60.6±8.5, 59.9±9.7 | 60.3±8.8 | NR | NR | 7.87±0.77, 7.92±0.72 | 7.90±0.72 | 0,0 | 1 |
| Bailey et al(45) | 2016 | NCT00528372 | 102weeks | DAPA 2.5mg,5mg,10mg | PLA | 65,64,70 | 75 | 53.0±11.7, 52.6±10.9, 50.6±10.0 | 52.7±10.3 | 2.1±3.2, 1.0±1.6, 2.3±3.7 | 2.1±3.1 | 7.92±0.9, 7.86±0.94, 8.01±0.96 | 7.84±0.87 | 0,0,1 | 1 |
| Hisamitsu Ishihara(46) | 2016 | NCT02175784 | 16weeks | IPRA 50mg | PLA | 175 | 87 | 58.7±11.1 | 59.2±9.3 | 12.59±7.79 | 14.28±8.54 | 8.67±0.77 | 8.62±0.86 | 4 | 1 |
| H W Rodbard(47) | 2016 | NR | 26weeks | CANA 100mg,300mg | PLA | 107 | 106 | 57.4±9.3 | 57.5±10.1 | 9.8±5.4 | 10.1±5.9 | 8.5±0.9 | 8.4±0.8 | 1 | 2 |
| Michael A Weber(48) | 2016 | NCT01195662 | 12weeks | DAPA 10mg | PLA | 225 | 224 | NR | NR | 7·7±5·9 | 7·3±5·0 | 8.1±0.9 | 8.0±1.0 | 1 | 0 |
| Wan Seman et al(49) | 2016 | NR | 12weeks | DAPA 10mg | SU | 58 | 52 | 53±9.1 | 56±9.1 | 5.0(3.0, 9.0)* | 6.0(3.0, 10.3)* | 7.7(7.08, 8.43)* | 7.6(6.9, 8.1)* | 11 | 5 |
| Weber et al(50) | 2016 | NCT01137474 | 12weeks | DAPA 10mg | PLA | 302 | 311 | 55.6±8.4 | 56.2±8.9 | 8.2±6.4 | 7.6±6.2 | 8.1±1.0 | 8.0±0.9 | 1 | 0 |
| Paola Fioretto(51) | 2018 | NCT02413398 | 24weeks | DAPA 10mg | PLA | 160 | 161 | NR | NR | 14.3±8.1 | 14.5±8.3 | 8.33±1.08 | 8.03±1.08 | 3 | 0 |
| Yutaka Seino(52) | 2018 | JapicCTI-142582 | 16weeks | LUSE 2.5mg | PLA | 159 | 74 | 57.4±10.3 | 57.1±10.9 | 11.7±7.6 | 12.1±6.8 | 8.70±0.83 | 8.84±0.83 | 7 | 1 |
| Yasuo Terauchi(53) | 2018 | NCT02201004 | 16weeks | TOFO 20mg | PLA | 141 | 70 | 59.1±10.8 | 56.4±10.0 | 15.02±9.36 | 12.39±7.34 | 8.53±0.75 | 8.40±0.65 | 11 | 2 |
| Wenying Yang(54) | 2018 | NCT02096705 | 24weeks | DAPA 10mg | PLA | 139 | 133 | 56.5±8.4 | 58.6±8.9 | 12.7±7.2 | 12.2±6.7 | 8.52±0.76 | 8.58±0.81 | 1 | 0 |
| Allegretti, A. S.(55) | 2019 | NCT02836873 | 24weeks | BEXA 20mg | PLA | 157 | 155 | 69.3±8.36 | 69.9±8.29 | 15.54±9.198 | 16.28±8.977 | 8.01±0.786 | 7.95±0.812 | 6 | 5 |
| Stephen D Wiviott(56) | 2019 | NCT01730534 | 201.6weeks | DAPA 10mg | PLA | 8582 | 8578 | 63.9±6.8 | 64±6.8 | NR | NR | NR | NR | 213 | 207 |
| Kenneth W Mahaffey(57) | 2019 | NCT02065791 | 26weeks | CANA 100mg | PLA | 2202 | 2199 | 62.85±8.95 | 63.15±9.15 | 15.55±8.65 | 16±8.55 | 8.25±1.3 | 8.3±1.3 | 144 | 115 |
| Carol Pollock(58) | 2019 | NCT02547935 | 24weeks | DAPA 10mg | PLA | 145 | 148 | 64.7±8.6 | 64.7±8.5 | 17.55±7.7 | 17.71±9.5 | 8.44±1.0 | 8.57±1.2 | 4 | 4 |
| Inoue et al(59) | 2019 | UMIN000018839 | 24weeks | IPRA 50mg | PLA | 24 | 24 | 60.5±9.8 | 60.8±12.1 | 15.9±7.7 | 19.1±10.7 | 8.12±0.93 | 8.30±0.65 | 1 | 0 |
| McMurray et al(60) | 2019 | NCT03036124 | 72.8weeks | DAPA 10mg | PLA | 2373 | 2371 | 66.2±11.0 | 66.5±10.8 | NR | NR | NR | NR | 2 | 5 |
| Milton Packer et al(61) | 2020 | NCT03057977 | 64weeks | EMPA 10mg | PLA | 1863 | 1867 | 67.2±10.8 | 66.5±11.2 | NR | NR | NR | NR | 197 | 184 |
| Matthew M Y Lee(62) | 2021 | NCT03485092 | 36 weeks | EMPA 10mg | PLA | 52 | 53 | 68.7±11.1 | 68.2±11.7 | 9.7±6.8 | 9.0±6.2 | 7.2±1.5 | 7.5±1.6 | 29 | 31 |
| Deepak L Bhatt(63) | 2021 | NCT03521934 | 36weeks | SOTA 200mg | PLA | 608 | 614 | 69(63–76)* | 70(64–76)* | NR | NR | 7.1(6.4–8.3)* | 7.2(6.4–8.2)* | 57 | 54 |
| Jonathan W Cunningham(64) | 2022 | NCT03619213 | 110.4weeks | DAPA 10mg | PLA | 3131 | 3132 | 71.8±9.6 | 71.5±9.5 | NR | NR | NR | NR | 42 | 32 |
|  |  |  |  |  |  |  |  |  |  |  |  |  |  |  |  |
| Data are number of patients(n)or mean (sd) unless stated otherwise; *Median (IQR) | | | | | |  |  |  |  |  |  |  |  |  |  |
| Abbreviation: BMI: body mass index; HbA1c: glycated hemoglobin; IQR: interquartile range; s.d.: standard deviation; DAPA: dapagliflozin; CANA: canagliflozin; IPRA: ipragliflozin; TOFO: tofogliflozin; | | | | | | | | | | | | | |  |  |
| EMPA: empagliflozin; BEXA:bexagliflozi;LUSE:luseogliflozin;SOTA:Sotagliflozin;PLA: placebo; GLIM: glimepiride;GLIP:glipizide;SITA: sitagliptin; SEMA:semaglutide;SU: sulphonylurea; NR: not report | | | | | | | | | | | |  |  |  |  |
