# Supplementary material for: Risk of hypovolemia associated with sodium–glucose cotransporter-2 inhibitors treatment: A meta-analysis of randomized controlled trials
Source: Front Cardiovasc Med. 2022 Nov 14;9:973129. doi: 10.3389/fcvm.2022.973129 (PMC9701837; doi:10.3389/fcvm.2022.973129)
Supplement: Supplementary file 3 [file Table_3.DOCX]

**Supplementary Material 3-Electronical Database search strategy**

**Embase query-4174 2022-10-04**

#1 diabetes AND mellitus OR (type AND 2 AND diabetes) OR (type AND ii AND diabetes)

#2 sodium AND glucose AND cotransporter AND 2 AND inhibitor OR (sodium AND glucose AND transporter AND 2 AND inhibitor*) OR (sodium AND glucose AND transporter AND ii AND inhibitor*) OR (sglt2 AND inhibitor*)

#3 canagliflozin OR dapagliflozin OR empagliflozin OR ertugliflozin OR tofogliflozin OR ipragliflozin OR remogliflozin

#4 randomi*ed AND controlled AND trial OR (randomi*ed AND trial)

#5 #2 OR #3

#6 #1 AND #4 AND #5

**CENTRAL query-2870 2022-10-04**

(Diabetes mellitus or type 2 diabetes or type ii diabetes):ti,ab,kw AND (sodium glucose transporter 2 inhibitor* or sodium glucose transporter ii inhibitor* or SGLT2 inhibitor or Canagliflozin or Dapagliflozin or Empagliflozin or Ertugliflozin or Tofogliflozin or Ipragliflozin or Remogliflozin):ti,ab,kw (Word variations have been searched)

**Pubmed query-2085 2022-10-04**

#20 Search ((((((((((((((Sodium glucose co-transporter) OR (((SGLT2) OR SGLT-2) OR SGLT 2)) OR ((((Tofogliflozin) OR Apleway) OR Deberza) OR CSG452)) OR ((Empagliflozin) OR Jardiance)) OR (((dapagliflozin) OR Farxiga) OR Forxiga)) OR ((Canagliflozin) OR Invokana)) OR ((Sotagliflozin) OR LX4211)) OR ((luseogliflozin) OR Lusefi)) OR ((ipragliflozin) OR Suglat)) OR ((remogliflozin) OR BHV091009)) OR ((sergliflozin) OR GW869682X)) OR (((ertugliflozin) OR MK-8835) OR PF-04971729))) AND (((random*) OR Randomized Controlled Trial[Publication Type]) OR ((RCT) OR RCTs))) AND (((Diabetes mellitus) OR type 2 diabetes) OR type ii diabetes)

#19 Search ((Diabetes mellitus) OR type 2 diabetes) OR type ii diabetes

#18 Search ((random*) OR Randomized Controlled Trial[Publication Type]) OR ((RCT) OR RCTs)

#17 Search (RCT) OR RCTs

#16 Search Randomized Controlled Trial[Publication Type]

#15 Search random*

#14 Search (((((((((((sodium glucose co-transporter) OR (((SGLT2) OR SGLT-2) OR sglt 2)) OR ((((tofogliflozin) OR apleway) OR deberza) OR CSG452)) OR ((empagliflozin) OR jardiance)) OR (((dapagliflozin) OR farxiga) OR forxiga)) OR ((canagliflozin) OR invokana)) OR ((sotagliflozin) OR LX4211)) OR ((luseogliflozin) OR lusefi)) OR ((ipragliflozin) OR suglat)) OR ((remo gliflozin) OR BHV091009)) OR ((sergliflozin) OR GW869682X)) OR (((ertugliflozin) OR MK-8835) OR PF-04971729)

#13 Search (((((((((((Sodium glucose co-transporter) OR (((SGLT2) OR SGLT-2) OR SGLT 2)) OR ((((Tofogliflozin) OR Apleway) OR Deberza) OR CSG452)) OR ((Empagliflozin) OR Jardiance)) OR (((dapagliflozin) OR Farxiga) OR Forxiga)) OR ((Canagliflozin) OR Invokana)) OR ((Sotagliflozin) OR LX4211)) OR ((luseogliflozin) OR Lusefi)) OR ((ipragliflozin) OR Suglat)) OR ((remogliflozin) OR BHV091009)) OR ((sergliflozin) OR GW869682X)) OR (((ertugliflozin) OR MK-8835) OR PF-04971729)

#12 Search ((ertugliflozin) OR MK-8835) OR PF-04971729

#11 Search (sergliflozin) OR GW869682X

#10 Search (remogliflozin) OR BHV091009

#9 Search (ipragliflozin) OR Suglat

#8 Search (luseogliflozin) OR Lusefi

#7 Search (Sotagliflozin) OR LX4211

#6 Search (Canagliflozin) OR Invokana

#5 Search ((dapagliflozin) OR Farxiga) OR Forxiga

#4 Search (Empagliflozin) OR Jardiance

#3 Search (((Tofogliflozin) OR Apleway) OR Deberza) OR CSG452

#2 Search ((SGLT2) OR SGLT-2) OR SGLT 2

#1 Search Sodium glucose co-transporter

**Scopus-154 2022-10-04**

( TITLE-ABS-KEY ( sodium  AND glucose  AND transporter  2  inhibitor*  OR  sodium  AND glucose  AND transporter  AND ii  AND inhibitor*  OR  sglt2  AND inhibitor )  AND  TITLE-ABS-KEY ( canagliflozin  OR  dapagliflozin  OR  empagliflozin  OR  ertugliflozin  OR  tofogliflozin  OR  ipragliflozin  OR  remogliflozin )  AND  TITLE-ABS-KEY ( diabetes  AND mellitus  OR  type  2  diabetes  OR  type  AND ii  AND diabetes ) )

**Web of science-2444 2022-10-04**

**#1 TS=(Sodium glucose co-transporter OR SGLT2 OR SGLT-2 OR SGLT 2 OR Tofogliflozin OR Apleway OR Deberza OR CSG452 OR Empagliflozin OR Jardiance OR dapagliflozin OR Farxiga OR Forxiga OR Canagliflozin OR Invokana OR Sotagliflozin OR LX4211 OR luseogliflozin OR Lusefi OR ipragliflozin OR Suglat OR remogliflozin OR BHV091009 OR sergliflozin OR GW869682X OR ertugliflozin OR MK-8835 OR PF-04971729)**

**#2 TS=(random* OR Randomized Controlled Trial OR RCT OR RCTs)**

**#3 TS=(Diabetes mellitus OR type 2 diabetes OR type ii diabetes)**

**#1 AND #2 AND #3**
