# Supplementary material for: Risk of hypovolemia associated with sodium–glucose cotransporter-2 inhibitors treatment: A meta-analysis of randomized controlled trials
Source: Front Cardiovasc Med. 2022 Nov 14;9:973129. doi: 10.3389/fcvm.2022.973129 (PMC9701837; doi:10.3389/fcvm.2022.973129)
Supplement: Supplementary file 4 [file Table_4.DOCX]

# Supplementary Material 1

## Lists of preferred terms

### Volume reduction (hypotension, dehydration, or hypovolemia)

Blood osmolarity increased, blood pressure ambulatory decreased, blood pressure decreased, blood pressure diastolic decreased, blood pressure immeasurable, blood pressure orthostatic abnormal, blood pressure orthostatic decreased, blood pressure systolic decreased, blood pressure systolic inspiratory decreased, blood urea nitrogen/creatinine ratio increased, capillary nail refill test abnormal, central venous pressure decreased, circulatory collapse, decreased ventricular preload, dehydration, diastolic hypotension, femoral pulse decreased, hypoperfusion, hypotension, hypovolaemic shock, left ventricular end-diastolic pressure decreased, mean arterial pressure decreased, orthostatic heart rate response increased, orthostatic hypotension, peripheral circulatory failure, pulmonary arterial pressure decreased, pulmonary arterial wedge pressure decreased, pulse volume decreased, radial pulse decreased, renal ischaemia, shock, syncope, urine flow decreased, urine output decreased, venous pressure decreased, venous pressure jugular decreased, volume blood decreased.
